# Electron beam induced deposition of silacyclohexane and dichlorosilacyclohexane: the role of dissociative ionization and dissociative electron attachment in the deposition process

**DOI:** 10.3762/bjnano.8.237

**Published:** 2017-11-10

**Authors:** Ragesh Kumar T P, Sangeetha Hari, Krishna K Damodaran, Oddur Ingólfsson, Cornelis W Hagen

**Affiliations:** 1 Department of Chemistry and Science Institute, University of Iceland, Dunhagi 3, 107 Reykjavik, Iceland; 2 Department of ImPhys, Delft University of Technology, Lorentzweg 1, 2628CJ Delft, Netherlands

**Keywords:** dichlorosilacyclohexane, dissociative electron attachment, dissociative ionization, electron beam induced deposition, low-energy electrons, silacyclohexane

## Abstract

We present first experiments on electron beam induced deposition of silacyclohexane (SCH) and dichlorosilacyclohexane (DCSCH) under a focused high-energy electron beam (FEBID). We compare the deposition dynamics observed when growing pillars of high aspect ratio from these compounds and we compare the proximity effect observed for these compounds. The two precursors show similar behaviour with regards to fragmentation through dissociative ionization in the gas phase under single-collision conditions. However, while DCSCH shows appreciable cross sections with regards to dissociative electron attachment, SCH is inert with respect to this process. We discuss our deposition experiments in context of the efficiency of these different electron-induced fragmentation processes. With regards to the deposition dynamics, we observe a substantially faster growth from DCSCH and a higher saturation diameter when growing pillars with high aspect ratio. However, both compounds show similar behaviour with regards to the proximity effect. With regards to the composition of the deposits, we observe that the C/Si ratio is similar for both compounds and in both cases close to the initial molecular stoichiometry. The oxygen content in the DCSCH deposits is about double that of the SCH deposits. Only marginal chlorine is observed in the deposits of from DCSCH. We discuss these observations in context of potential approaches for Si deposition.

## Introduction

Focused electron beam induced deposition (FEBID) [1–2] is a 3-D direct writing method suitable for the fabrication of nanostructures, even on non-planar surfaces. This approach is in many ways complementary to current mask-based lithography methods and has high potential in areas where these are not applicable. Focused electron beam induced deposition is based on the exposure of precursor molecules, physisorbed on a substrates surface, to a narrowly focused high-energy electron beam. Ideally these precursor molecules fully decompose under the electron beam and a well-defined deposit is formed from the non-volatile fragments while the volatiles are pumped away. The ideal case would be that the primary electron beam alone is responsible for the decomposition of these molecules through effective impulsive energy transfer. Then, the decomposition of the precursor molecules would be confined within the diameter of the primary electron beam and a spatial resolution better than 1 nm would be achievable on a routine basis.

However, when a high-energy electron beam impinges on a solid substrate, significant inelastic and elastic scattering will take place at the surface and within the substrate along the penetration depth of the beam [3–4]. Furthermore, a significant number of secondary electrons are produced through inelastic ionizing scattering of the primary beam and its scattered electrons [3]. On a flat surface the spatial distribution of these secondary electrons will be defined by the angular distribution of the back-scattered primary electrons [5–7]. During the growth of structures with aspect ratios greater than zero, however, the forward component will also play a role, generating a flux of secondary electrons on the surface of objects with high aspect ratio as these are grown [8–9]. The energy distribution of the secondary electrons produced depends largely on the nature of the substrate [10–11], but also on the primary electron energy. However, it normally has similar features: a maximum well below 10 eV with still a significant contribution close to 0 eV and a high-energy tail extending well above 100 eV [3,5,12]. In this energy range electron induced molecular fragmentation may proceed through four different processes: dissociative electron attachment (DEA; Equation 1), dissociative ionization (DI; Equation 2), neutral dissociation (ND; Equation 3) and dipolar dissociation (DD; Equation 4) [13–20]. The respective reaction schemes for each of these pathways are:

[1]
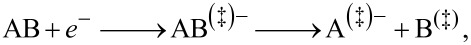


[2]
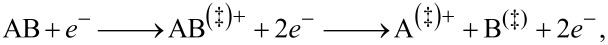


[3]
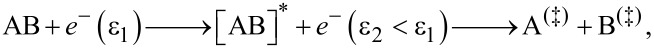


[4]
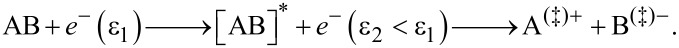


The double dagger (‡) signifies vibrational or electronic excitation, the asterisk identifies electronically excited species and ε_1_ and ε_2_ are the energies of the electron before and after the inelastic scattering event, respectively. These reactions have very different energy dependencies, their cross sections also have different dependencies on the respective molecular constellation, and the product formation through these channels is very different.

In recent years significant, concerted effort has been taken to de-convolute the effect of these different processes to better understand the physics and chemistry behind the FEBID process and to purposely turn that knowledge into applicable design criteria for superior FEBID precursors. In this context a considerable number of gas-phase studies have been conducted, mainly on DEA and DI of different organometallic FEBID precursors. Complementary surface science studies have been carried out to better relate the gas-phase observations to the actual conditions in FEBID. A fairly comprehensive account of these studies up to early 2015 is given in [13]. This is however a fast-moving field and a considerable number of studies have appeared recently [21–29], including studies on a mononuclear heteroleptic precursor [21,24–26] and on large heteronuclear carbonyl cluster compounds [22–23,30] that have partly proven to perform well in the FEBID deposition of magnetic alloys [31]. In fact, both DEA and DI cross sections of typical metal-containing FEBID precursors can be very high [32–33]. The same is true for electronic excitation upon electron impact [34]. However, no experimental information is available on actual cross sections for neutral dissociation upon such electronic excitations. This is due to the difficulties associated with the detection of the resulting neutral species and current experiments are thus largely confined to DEA and DI of FEBID precursors. Despite this, significant insight has been provided by the gas-phase and surface-science studies and in individual cases a distinction between the role of DEA and DI in the deposition process has been achieved.

**Figure 1:**
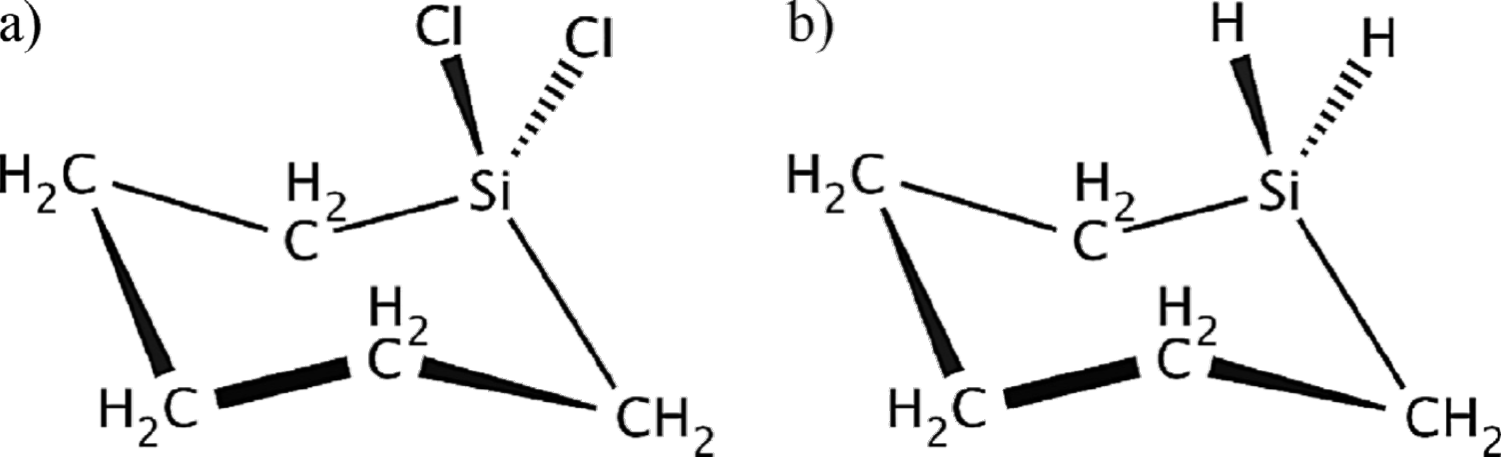
Molecular structure of (a) 1,1-dichloro-1-silacyclohexane (cyclo-C_5_H_10_SiCl_2_) and (b) silacyclohexane (cyclo-C_5_H_10_SiH_2_). Adapted from [35].

Silacyclohexane (SCH) and dichlorosilacyclohexane (DSCH), shown in Figure 1, are cyclohexane derivatives where one of the carbon atoms is replaced by a silicon atom, and in DCSCH two chlorine atoms are attached to that silicon atom.

**Figure 2:**
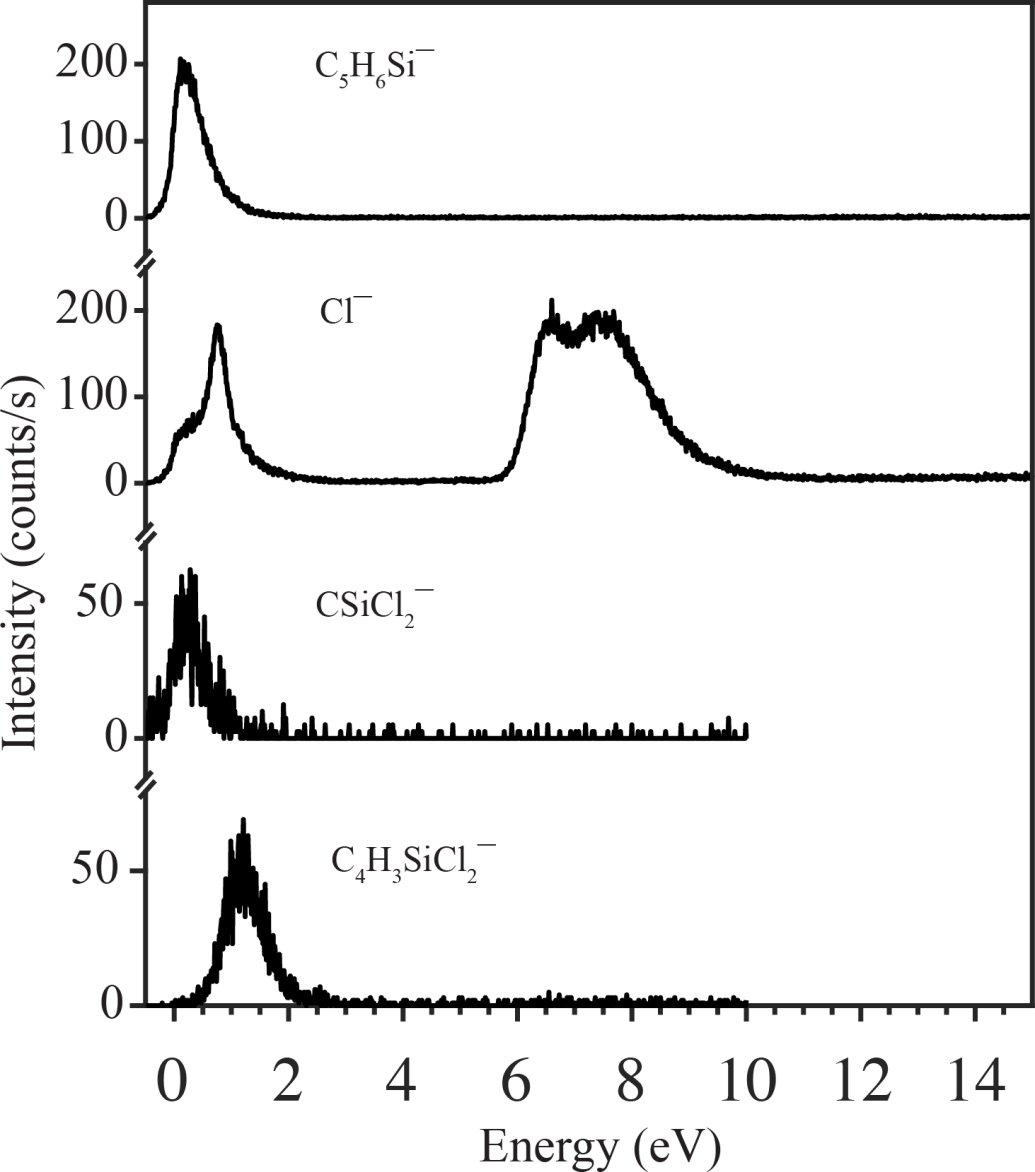
Negative ion yield curve for the principal fragments formed by the electron attachment dissociation of DCSCH in the energy range from 0–14 eV. Adapted from [35].

**Figure 3:**
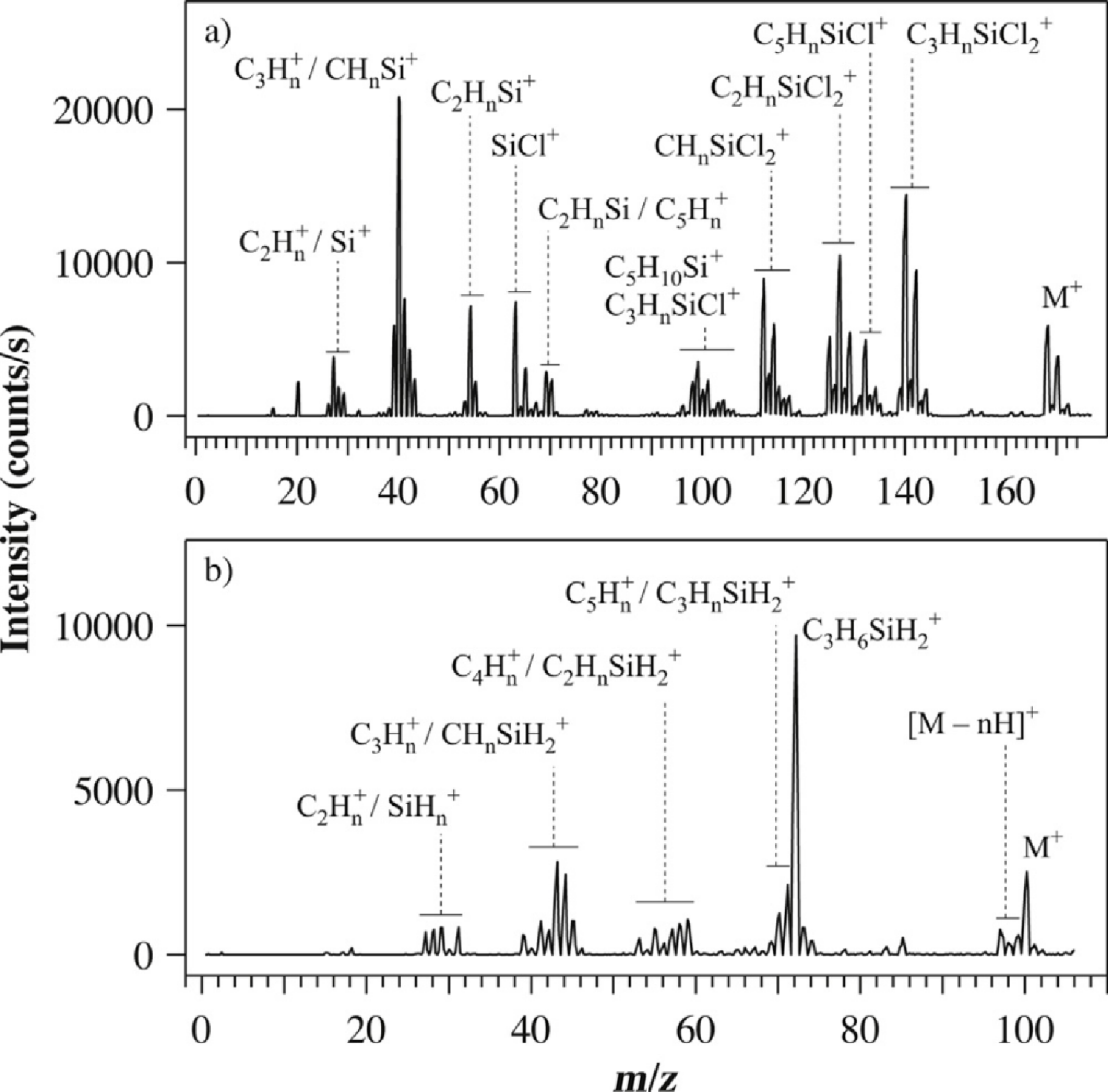
Positive ion mass spectra of (a) DCSCH and (b) SCH, both spectra are recorded at an electron impact energy of 70 eV. Adapted from [35].

In a fairly recent gas phase study [35], where these molecules were exposed to low-energy electrons under single-collision conditions, it was shown that while appreciable decomposition of DCSCH was affected through DEA, SCH was inert with regards to this process. Dissociative ionisation, on the other hand, leads to similar fragmentation of both these molecules. For reference, Figure 2 shows the ion yield curves for the principal DEA channels observed for DCSCH and Figure 3 compares the DI spectra for DCSCH and SCH at an electron impact energy of 70 eV.

Motivated by the absolute difference in the sensitivity of these compounds towards fragmentation induced by DEA, we have conducted the first EBID experiments with DCSCH and SCH, and we discuss these in the context of potential effects on the growth dynamics through the very different sensitivity of these molecules towards electrons of very low energy (<10 eV). Furthermore, both molecules are potential precursors for the deposition of SiO_2_, especially in conjunction with oxidizing agents such as oxygen or water. Specifically such deposits are of interest due to the broad transparency and the high diffractive index of SiO_2_, but may also be of interest in the fabrication of protective or isolating layers/components [2]. Specifically, FEBID deposition of SiO_2_ is of interest for the repair of deep ultraviolet (DUV) masks [36], but also for the deposition of transparent nano-optics [37–39].

## Results and Discussion

### Deposition from SCH and DCSCH

**Figure 4:**
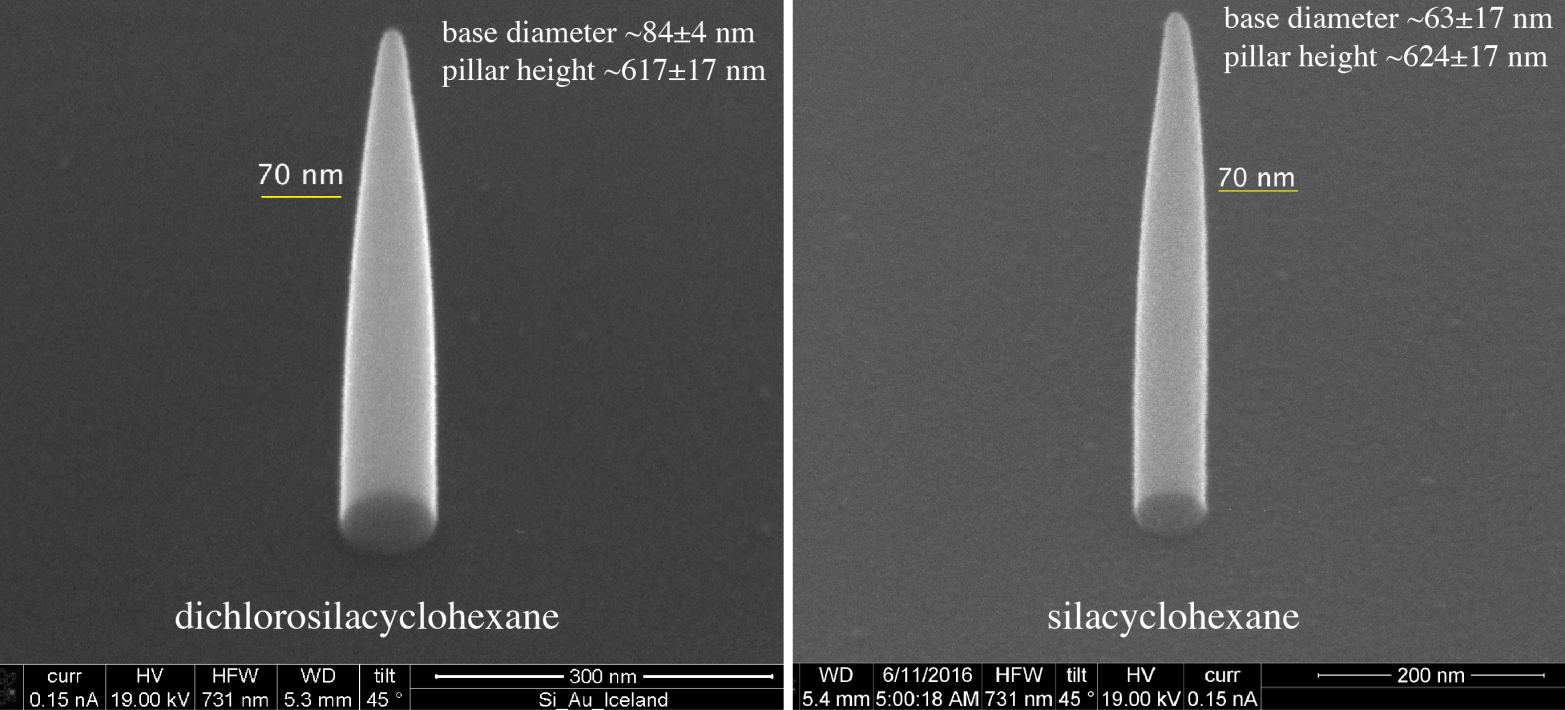
Pillars grown by EBID from the precursors DCSCH (left) and SCH (right). The precursor pressure was 3 × 10^−5^ mbar and the total deposition time was 180 s for both DCSCH and SCH. Electron beam energy of 20 keV and current of 150 pA was used for the deposition. The base diameters and heights are given in the images.

**Figure 5:**
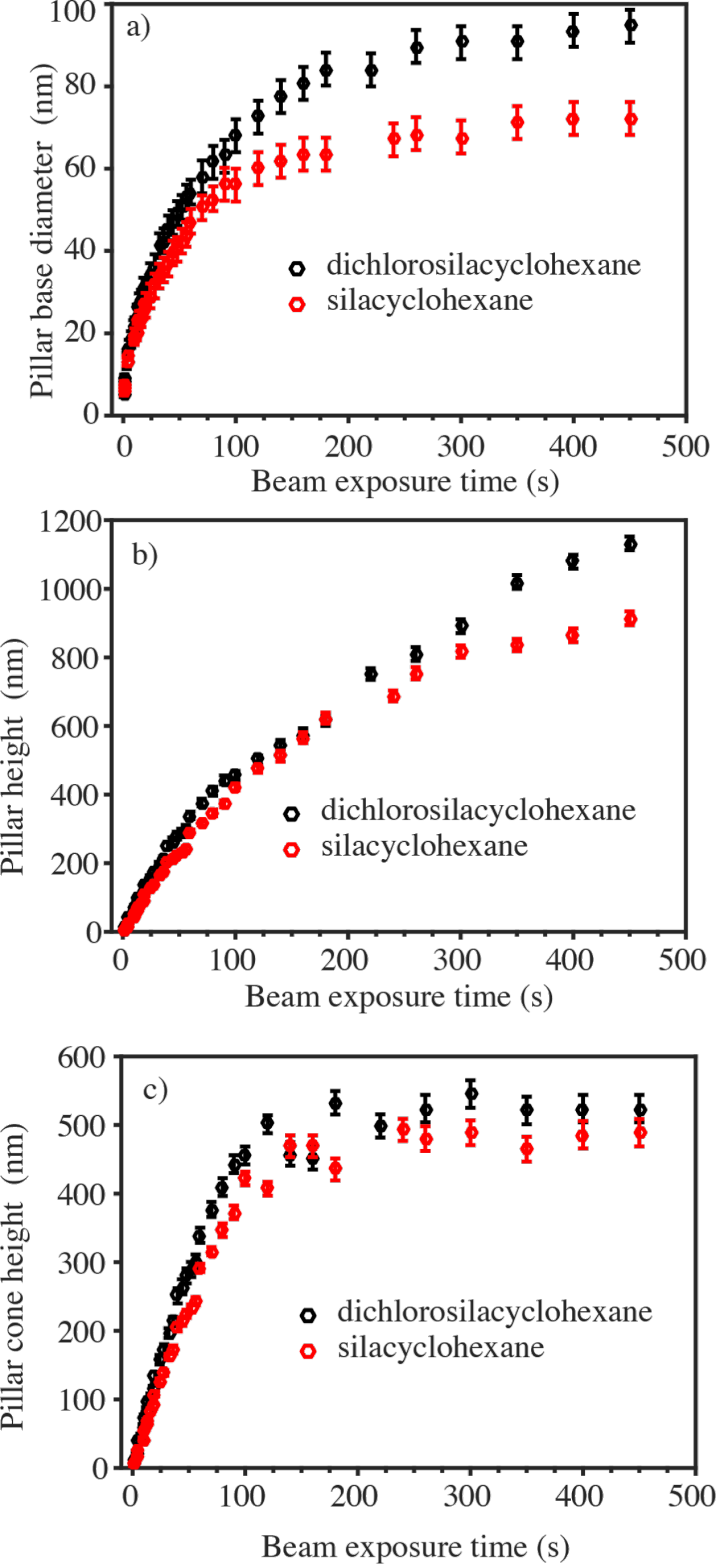
Measured (a) pillar base diameter, (b) pillar height and (c) heights of the cone-shaped upper part of the pillars as a function of electron beam exposure time for two precursors DCSCH and SCH as black and red circles, respectively. The experimental parameters such as precursor pressure, electron beam energy, current were kept constant during the experiment.

To our knowledge, the precursor molecules SCH and DCSCH have not been used for EBID so far. Hence, the first experiment that was performed was just to observe whether something can be deposited from each of these precursor molecules. The precursor was introduced via a leak valve into the specimen chamber of a scanning electron microscope (SEM) (see Experimental section for details), raising the pressure from below 9 × 10^−7^ mbar to (2–3) × 10^−5^ mbar. Subsequently, an electron beam was focused on a silicon substrate and it was observed whether or not a pillar grows under a stationary electron beam. Both precursor molecules were seen to easily dissociate and to form solid deposits. Figure 4 shows two pillars grown from DCSCH (left) and SCH (right) at the same precursor pressure of ca. 3 × 10^−5^ mbar and with the same total deposition time of 180 s. The first observation is that the height of both pillars is about the same but the SCH pillar has a smaller base diameter than the DCSCH pillar. Also, both pillars are characterised by a cylindrical lower part and a conical upper part. To study the growth characteristics of both precursors, pillars were grown for a range of deposition times, keeping all other parameters, such as precursor pressure, beam energy and beam current, the same. Figure 5a shows the pillar base diameter as a function of the total beam exposure time for SCH and DCSCH. The diameters were measured from the SEM images, as described in the Experimental section. Pillars deposited with a beam exposure time below 600 ms (SCH) and below 300 ms (DCSCH) could hardly be imaged anymore. Therefore those pillar diameters are not included in Figure 5. For both precursors an abrupt increase in pillar base diameter is seen at the initial growth stage, and after about 300 s the pillar base diameter starts saturating. The highest initial lateral growth rate we could measure for DCSCH and SCH was 12 nm/s (measured at 300 ms) and 8 nm/s (measured at 600 ms beam exposure time). After 300 s, the lateral growth rate decreases significantly and saturates at a base diameter of ca. 90 nm and ca. 70 nm, respectively. The diameters of the DCSCH pillars are larger than those of the SCH pillars over the entire range of deposition times. Figure 5b shows how the pillar height develops for increasing deposition time for both precursor molecules. Both curves show a linearly increasing height for small exposure times and a slightly decreasing vertical growth rate at higher exposure times.

The composition of the deposits was determined using energy-dispersive X-ray analysis (EDX) on large and thick deposits. The deposits were grown on a gold sample to be able to distinguish the elements in the deposit from the substrate material.

For the precursor molecule DCSCH, respectively SCH, measurements at four, respectively three, different sites were performed, the results of which were averaged. The ratio of Si/O/C in the SCH deposits was found to be 1.0/1.1/6.0. The ratio of Si/O/C/Cl in the DCSCH deposits was found to be 1.0/2.4/5.8/0.2. In both cases the Si/C ratio is close to that of the precursor molecules (1/5) and the additional carbon content is likely to result from the background gas. No significant chlorine content is observed for DCSCH. However, the oxide content in the deposits formed from DCSCH is significantly larger than that in deposits from SCH. In fact, the Si/O ratio from DCSCH is 1/2.4 indicating a complete oxidation to SiO_2_. For SCH this ratio is only 1/1.2 indicating a much more incomplete oxidation. Silicon chlorides are generally very sensitive towards hydrolysis leading to the formation of silicon oxide and hydrochloric acid. We thus anticipate that the significantly larger oxygen content in the deposits formed from DCSCH is the result of hydrolysis in reactions with residual water in the background gas and at the surfaces.

In EBID pillar growth one can distinguish three stages: the nucleation stage, a fast-growth stage and a saturation stage [4,40]. In the nucleation stage a dot-like deposit will form, predominantly due to the scattered electrons emitted from the substrate surface. In the fast-growth stage, a cone shaped pillar grows with maximum lateral and vertical growth rate. During the fast growth stage, the growth is enhanced by forward- and backscattered electrons (FSEs and BSEs) generated in the growing deposit, as well as by secondary electrons (SE2s) created by FSEs + BSEs [40]. The evolution of the cone morphology depends upon the spatial extent and the location of the primary electron beam interaction volume, and also depends on the material [40]. Eventually, when the interaction volume is limited to the pillar volume the lateral growth saturates. The cone will no longer change shape and the pillar will grow taller in a cylindrical fashion. The cone angles measured from SEM tilt images at the beginning stage of pillar growth for DCSCH and SCH were 71° and 74°. After 80 to 100 s, the angles reduce to a constant value of 13° and 11°, respectively. In Figure 5c the heights of the cone-shaped upper parts of the pillars deposited with SCH and DCSCH are shown for varying exposure times. It is seen that the tip cone height for DCSCH and SCH saturates at about 500 nm. This value is significantly lower than the spatial extent of the interaction volume of bulk Si. Using the equation of Kayana and Okayama [41] a value for the spatial extent of the interaction volume in Si is estimated as 4.7 μm (at 20 keV). However, the size of the interaction volume in a pillar does not necessarily have to be the same as in the bulk, because of the reduced scattering in a pillar. For example, the Monte Carlo simulated mean electron penetration depth for 20 keV electrons in a flat aluminium substrate is 3200 nm [42] while the simulated electron penetration depth for 20 keV electrons in a pillar with a cone angle of 10° is only 240 nm [42]. Similarly, the simulated depth of the interaction volume for 20 keV electrons in bulk SiO_2_ is ca. 3 μm [43], while the calculated averaged depth of the interaction volume for 20 keV electrons in a 350 nm pillar is ca. 500 nm [43]. Assuming that the EBID pillars consist of SiO_2_ (ignoring the large carbon content found) the interaction volume for an 80 nm SiO_2_ pillar might be below 500 nm, in agreement with the tip cone height saturation value of Figure 5c. At exposure times larger than 180 s the lower part of the pillars grows in a cylindrical shape. Since the pillar base diameter (Figure 5a) and the tip cone height (Figure 5c) saturate at about 180 s for DCSCH and SCH, one could define this as the saturation region, where the lateral growth rate diminishes to zero but the pillar height keeps increasing.

**Figure 6:**
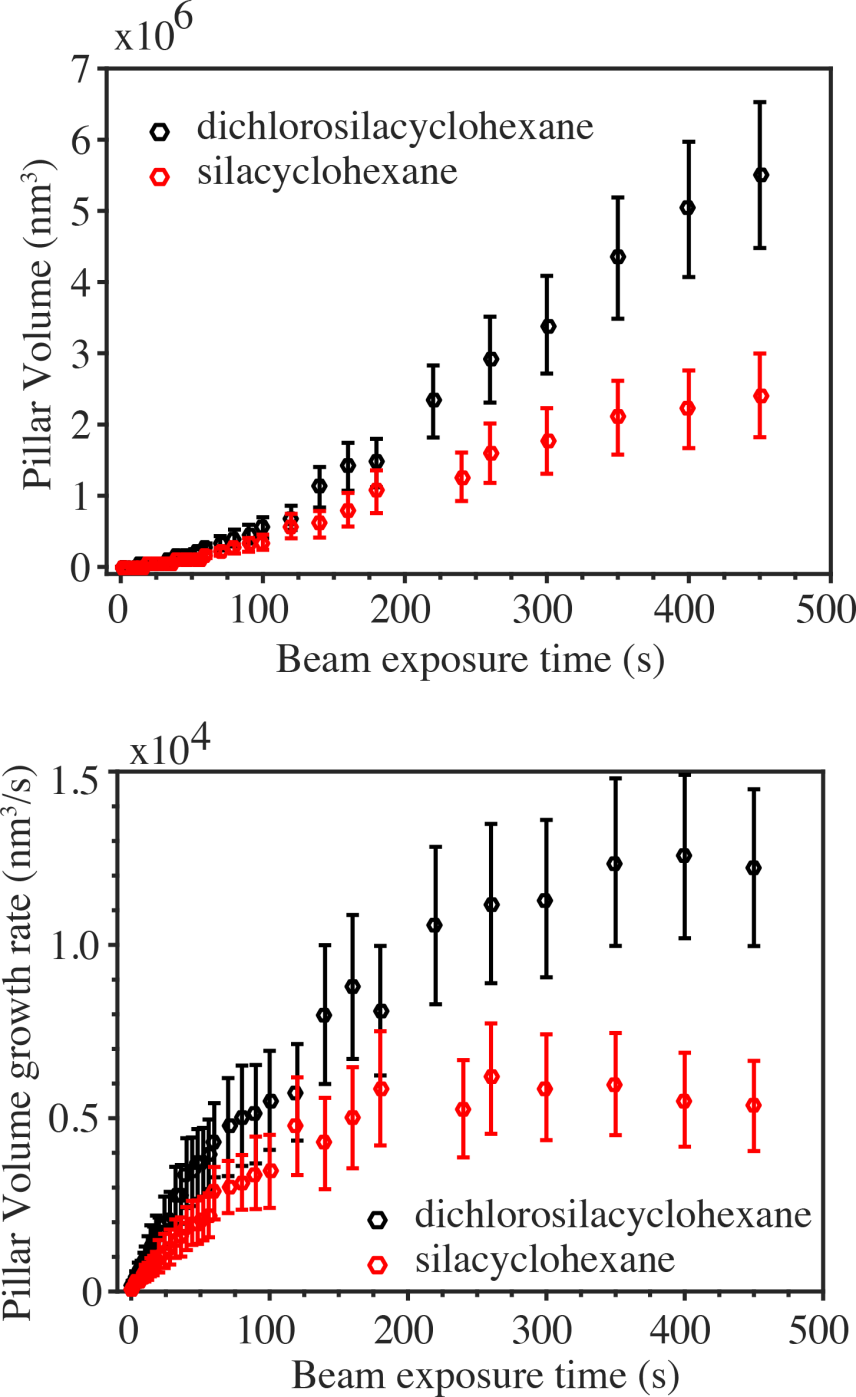
(a) Pillar volume determined from the measured pillar diameter, pillar height, tip cone height and cone angle, versus exposure time for DCSCH and SCH. (b) the pillar volume growth rate versus exposure time for DCSCH and CSH.

From the measured pillar dimensions the pillar volume can now be estimated. Figure 6a shows the pillar volume as a function of the exposure time for SCH and DCSCH, the volume being larger for DCSCH over the entire range. Interestingly, a closer inspection of the volume increases at exposure times below 100 s reveals that the growth is quadratic rather than linear. This becomes better visible looking at the volume growth rate, which is plotted in Figure 6b, showing a significantly larger growth rate for DCSCH than for SCH.

A linear increase in pillar height with beam exposure time [43–46] is usually taken as an indication that the deposition is carried out in the electron-limited regime, i.e., there is always sufficient coverage of the surface with precursor molecules. This is also observed for DCSCH and SCH pillars. However, the pillar volume growth is faster, and rather quadratic than linear with exposure time. Were the deposition process in the precursor-limited regime, a slower growth with exposure time would be expected. As the deposits may be insulating or, at best, be very bad electrical conductors, electron-induced heating of the pillars may occur. This would lower the residence time of precursor molecules, and thereby decrease the growth rate. This is, for instance, observed at larger exposure times in the experiments. Also surface diffusion of the precursor molecules would rather slow down the growth with increasing exposure time than increase the growth.

Charging could play a role in case the deposits are insulating, although it is not straightforward to predict its influence on the volume growth, and no conclusive explanation for the increasing volume growth rate can be offered yet.

Comparing the lateral growth of DCSCH and SCH pillars below 180 s exposure time, the DCSCH pillars were seen to have a larger base diameter than the SCH pillars. The difference in diameter is ca. 1.3 nm in the beginning and gradually increases with beam exposure time to ca. 20 nm at 180 s. From Figure 6b it is seen that the volume growth rate of DCSCH is twice that of SCH, in the early growth stage and the same enhancement in volume growth rate of DCSCH occurs at higher beam exposure times (i.e., DCSCH volume growth rate is about two times that of SCH). As the pillar diameter is determined mainly by the FSEs and BSEs and the SE2s [15], one could expect a smaller growth in pillar width when DEA channels are not available, as is the case for SCH.

From the DEA study of DCSCH (Figure 2 and [35]), one can see that DEA is mainly active below 2 eV, and in the range of 6–9 eV, with the integral cross section being similar for both these energy ranges. This means that the inert behaviour of SCH towards DEA, as compared to DCSCH, only concerns electrons of energies below 2 eV and in the range of 6–9 eV. The effective dissociation yield of DEA in the DCSCH EBID process, however, depends not only on the DEA cross sections, but also on the available number of electrons within the respective energy ranges. From the secondary electron emission spectra of Si irradiated at 1 keV [47], the integrated contribution of emitted electrons with energies below 2 eV and in the energy range from 6 to 9 eV can be estimated to be close to 50% of the total emitted SEs below 20 eV and extrapolation of the secondary electron yield to 100 eV would lower this value to some extent. Since we do not have estimates of the DEA and DI cross sections for DCSCH and SCH, it is difficult to know how much DEA contributes to the deposition compared to DI. In the early pillar-growth stage, the difference in pillar base diameter between DCSCH and SCH is still small. But at later stages the difference increases and grows to a maximum at the saturation point (i.e., at 180 s). At this saturation point, the SE yield will be maximum as shown in [9,40], and the observed diameter difference might be attributed to the electrons below 2 eV and in the range of 6–9 eV if they amount to 30% of all SE events, with the other effects being the same for both compounds.

In summary, DEA would cause additional lateral growth and a higher volume growth rate for DCSCH compared to SCH, but insufficient evidence is obtained to fully ascribe the observed additional lateral growth to this effect only. The electron scattering in deposits from DCSCH and SCH may be different, resulting in different electron yields. Also, the two types of precursor molecules may behave differently when introduced in the specimen chamber, e.g., they may have different sticking coefficients and different surface densities.

In the next section, experiments are presented where deposits are grown in close proximity of each other to further study the role of the low-energy secondary electrons in EBID using DCSCH and SCH as precursors.

### Proximity effect comparison between DCSCH and SCH precursors

**Figure 7:**
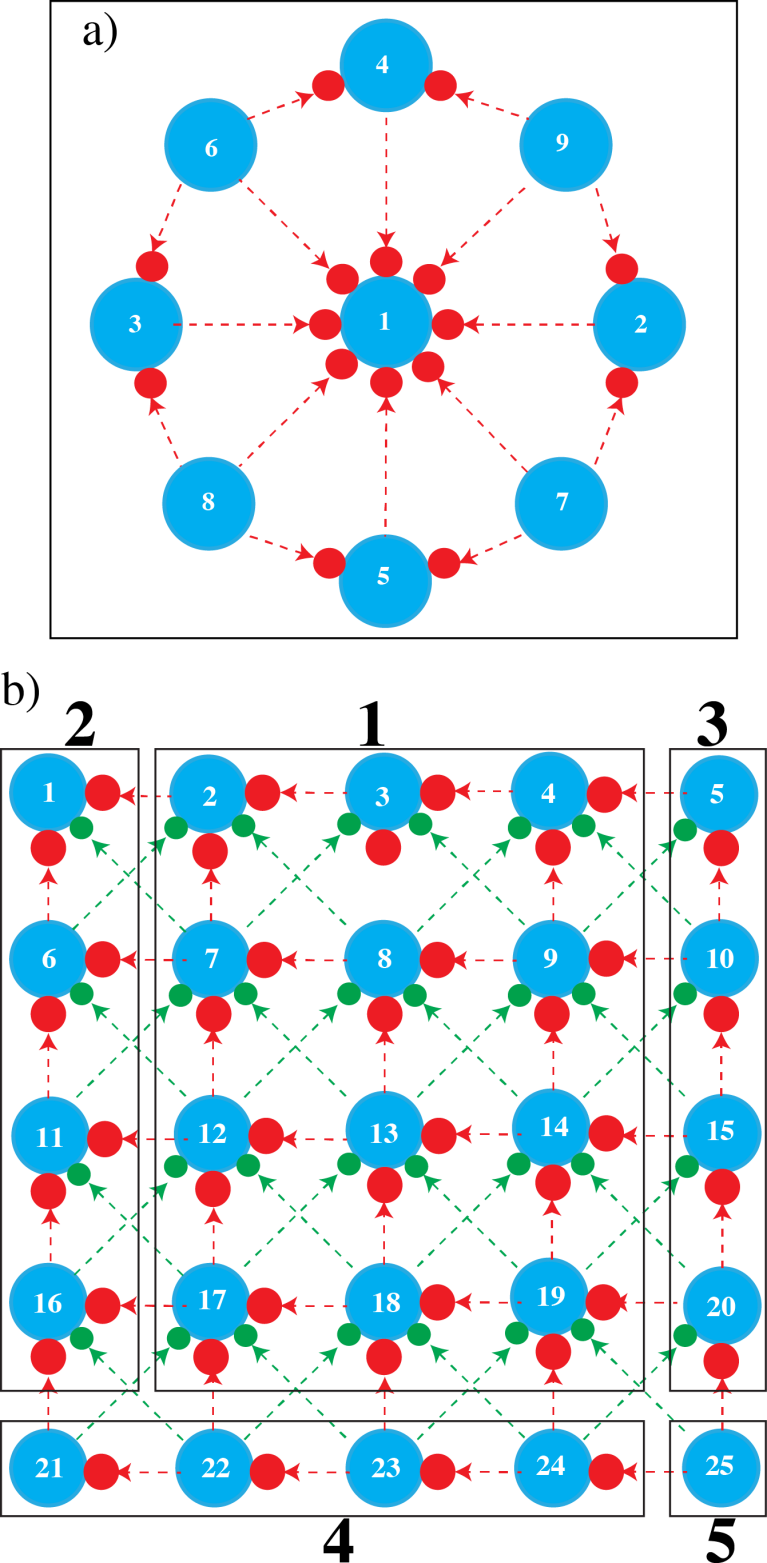
a) Schematic showing the order of pillar deposition in a circular array around a central dot; b) schematic showing the order of pillar deposition in a square array. Blue filled circles represent the diameter of the pillars with no proximity effect, red filled circles represent the lateral broadening induced by the deposition of pillars in close proximity (nearest neighbours) and green filled circles represent the lateral broadening induced by the deposition of pillars that are not as close, but still capable of inducing additional deposition. In the circular configuration these were not indicated and in the square configuration these are the next diagonal neighbours. The bold numbers, shown for the square configuration, represent the areas with the same expected pillar diameters.

When growing a pillar close to an earlier deposited pillar, SEs and BSEs + FSEs and SE2s are emitted from the newly deposited pillar, inducing additional deposition on the earlier deposited pillar. This is called a proximity effect and it is believed that, especially for shallow deposits in the early stage of growth, the proximity effect is predominantly caused by the low-energy SEs emitted from the neighbouring deposit. Therefore, the presence or absence of effective DEA channels in the precursor dissociation may be reflected in the extent of the proximity effect. The proximity effect is usually observed as a variation of dot diameters within an array of deposited dots, depending on the order in which they are deposited. Dots were deposited from DCSCH and SCH in two different geometries. In Figure 7a, a schematic is shown for a circular arrangement of dots. The first pillar is deposited in the centre of a circle and the other pillars are deposited surrounding the central pillar. The order in which they are deposited is indicated in Figure 7a as 1 to 9. The expected additional broadening due to the proximity effect is indicated in the schematic by the red filled circles. The red arrows indicate the pillars causing the additional deposition. The number of red circles around the blue dots gives an impression of the expected broadening when proximity effects are present. In the absence of proximity effects, all dots are expected to have almost the same diameter after deposition. In Figure 7b, a different arrangement is shown in which dots are deposited in a square array. The blue filled circles represent the pillars with no additional broadening and the numbers in the blue filled circles indicate the order of pillar deposition. The red and green filled circles represent the expected additional deposition due to the proximity effect and the red and green arrows indicate the origin of the effect. For example, pillar 2 can have additional deposition during the deposition of pillar 3 (red arrow), pillar 7 (red arrow), pillar 8 (green arrow) and pillar 6 (green arrow).

From the drawing in Figure 7a, it is seen that in the presence of proximity effects, the largest dot is expected to be dot 1, the second largest dots should be 2–5, and the smallest dots should be 6–9. Similarly, in Figure 7b areas where similar diameters are expected are grouped, indicated by the areas numbered from 1 to 5. The broadening due to the proximity effect should decrease from area 1 to 5. All pillars were grown very close to each other in an area much smaller than the backscattered electron range of Si at 20 keV (4–5 micrometres), such that the backscattered electrons of the substrate have approximately the same effect on all dots.

**Figure 8:**
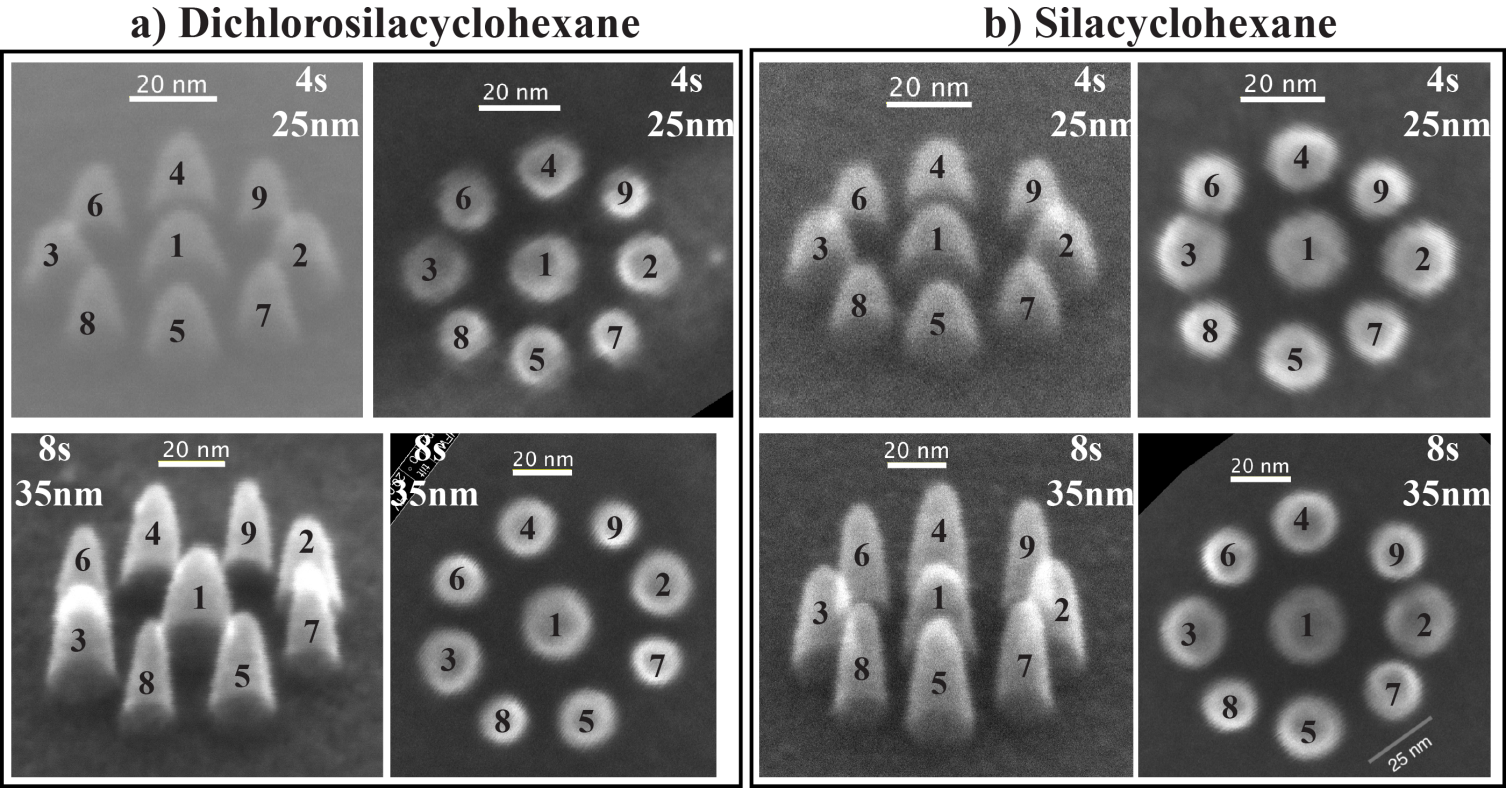
Tilted (45°) and normal view of two sets of nine closely spaced pillars deposited in a circular arrangement; (a) deposited with DCSCH and (b) deposited with SCH. The beam exposure time and the distance between nearby pillars are marked in the corner of each image.

Figure 8a shows two sets of nine closely spaced pillars deposited with DCSCH in a circular arrangement. Both a top-down SEM image and a 45° tilt image are shown. The beam exposure time and the separation between the central pillar and the surrounding pillars are given in the figure. Figure 8b shows the dots deposited with SCH, all other parameters are exactly the same as for Figure 8a. The diameters of all pillars deposited in the circular arrangement are tabulated in Table 1. It is clearly seen in both figures that a proximity effect is present. As expected, dots 2–5 and dots 6–9 indeed have almost the same diameter, and the order from small to large is as predicted. The dot base plane diameters were measured as described in the Experimental section.

**Table 1:** Measured pillar base diameter (nm) of DCSCH and SCH pillars deposited in a circular arrangement. The method for measuring the pillar base diameter is described in the Experimental section.

	DCSCH				SCH			
	4 s, 25 nm		8 s, 35 nm		4 s, 25 nm		8 s, 35 nm	
pillar	45° view	normal view	45° view	normal view	45° view	normal view	45° view	normal view

1	19.2 ± 4	16.5 ± 4	24.6 ± 4	22.5 ± 4	19.7 ± 4	17.9 ± 4	26.1 ± 4	24.5 ± 4
2	15.0 ± 4	14.5 ± 4	20.8 ± 4	19.5 ± 4	17.2 ± 4	16.0 ± 4	23.7 ± 4	21.5 ± 4
3	16.3 ± 4	15.2 ± 4	21.0 ± 4	19.5 ± 4	16.0 ± 4	16.1 ± 4	23.7 ± 4	21.2 ± 4
4	14.5 ± 4	14.9 ± 4	18.3 ± 4	18.4 ± 4	15.9 ± 4	15.4 ± 4	22.0 ± 4	20.2 ± 4
5	16.0 ± 4	13.4 ± 4	20.5 ± 4	18.0 ± 4	16.9 ± 4	14.8 ± 4	23.0 ± 4	20.7 ± 4

6	13.2 ± 2	13.0 ± 2	16.0 ± 2	15.4 ± 2	14.4 ± 2	13.4 ± 2	19.7 ± 2	18.2 ± 2
7	13.8 ± 2	11.2 ± 2	17.3 ± 2	15.0 ± 2	14.7 ± 2	13.6 ± 2	18.7 ± 2	17.9 ± 2
8	13.2 ± 2	11.2 ± 2	15.3 ± 2	14.5 ± 2	14.7 ± 2	12.8 ± 2	18.7 ± 2	17.5 ± 2
9	12.2 ± 2	11.0 ± 2	15.4 ± 2	15.0 ± 2	13.9 ± 2	13.2 ± 2	17.7 ± 2	17.5 ± 2

In the case of DCSCH, the first pillar deposited in a circular arrangement, with a beam exposure time of 4 s, has a diameter of 17.9 ± 3 nm, the average diameter of pillars 2 to 5, with the same beam exposure time, is 15.0 ± 1 nm, for pillars 6 to 9 an average diameter of 12.4 ± 1 nm is observed. A similar analysis of the SCH deposits results in a diameter of 18.8 ± 3 nm for the first deposited pillar, the pillars 2–5 have an average diameter of 16.0 ± 1 nm, and pillars 6–9 have an average diameter of 13.8 ± 1 nm. The observed relative broadening of the central pillar with respect to the average diameter of pillars 6–9 is 5.5 ± 1 nm for DCSCH, and 5.0 ± 1 nm for SCH. For an exposure time of 8 s the same analysis results in a relative broadening for DCSCH and SCH of 8.0 ± 1 and 7.7 ± 1 nm, respectively.

**Figure 9:**
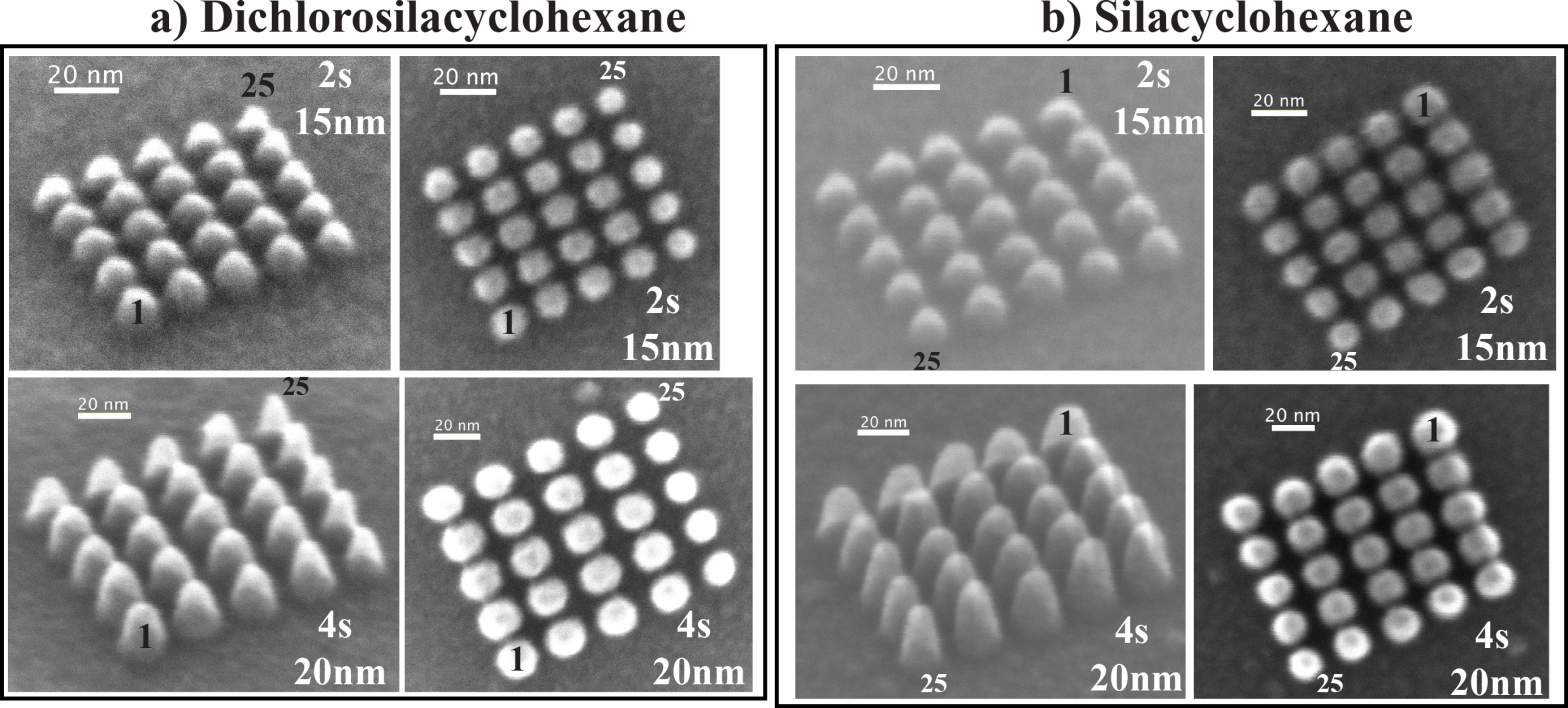
Square arrays of pillars deposited with the indicated beam exposure time and neighbouring pillar distance from (a) DCSCH and (b) SCH. The first (1) and last (25) pillars are indicated and the order of deposition is as shown in Figure 7b.

The square arrangement of deposited pillars is shown in Figure 9. Two sets of square arrays of pillars are shown, for both DCSCH and SCH. The first set is deposited with a beam exposure time of 2 s and the distance between the pillars is 15 nm. In the second set, pillars were deposited with a beam exposure time of 4 s and the distance between the pillars is 20 nm. The order of deposition is the same as in the schematic shown in Figure 7b. The diameters of all pillars deposited in the square arrangement are tabulated in Table 2 along with the average diameter within each section.

**Table 2:** Measured pillar base diameter (nm) of DCSCH and SCH pillars deposited in a square array. There are five different areas in the square array within which the pillars are expected to have the same base diameter. The average pillar base diameter is shown at the bottom row of each area. The method for measuring the pillar base diameter is described in the Experimental section.

		DCSCH		SCH	
	pillar	2 s, 15 nm	4 s, 20 nm	2 s, 15 nm	4 s, 20 nm

area 1	2	12.8 ± 4	16.8 ± 4	13.5 ± 4	18.4 ± 4
	3	12.3 ± 4	16.3 ± 4	13.6 ± 4	18.6 ± 4
	4	12.5 ± 4	17.4 ± 4	12.9 ± 4	17.9 ± 4
	7	12.0 ± 4	16.0 ± 4	12.5 ± 4	16.3 ± 4
	8	12.6 ± 4	16.1 ± 4	12.3 ± 4	17.4 ± 4
	9	12.6 ± 4	16.1 ± 4	12.4 ± 4	16.5 ± 4
	12	12.0 ± 4	16.3 ± 4	12.1 ± 4	16.4 ± 4
	13	11.9 ± 4	16.4 ± 4	12.2 ± 4	16.9 ± 4
	14	11.9 ± 4	16.1 ± 4	12.0 ± 4	16.0 ± 4
	17	11.9 ± 4	16.1 ± 4	12.3 ± 4	17.0 ± 4
	18	11.6 ± 4	15.9 ± 4	11.9 ± 4	16.5 ± 4
	19	11.5 ± 4	15.8 ± 4	12.0 ± 4	16.5 ± 4

area 1 average pillar diameter		12.1 ± 1	16.3 ± 1	12.5 ± 1	17.0 ± 1

area 2	1	13.4 ± 4	16.9 ± 4	13.9 ± 4	19.6 ± 4
	6	12.3 ± 4	16.5 ± 4	12.7 ± 4	18.5 ± 4
	11	12.1 ± 4	16.3 ± 4	13.0 ± 4	18.6 ± 4
	16	11.7 ± 4	15.8 ± 4	12.8 ± 4	17.4 ± 4

area 2 average pillar diameter		12.4 ± 2	16.4 ± 2	13.1 ± 2	18.5 ± 2

area 3	5	12.5 ± 2	15.6 ± 2	11.9 ± 2	16.4 ± 2
	10	11.9 ± 2	15.3 ± 2	11.9 ± 2	16.6 ± 2
	15	11.4 ± 2	14.9 ± 2	11.8 ± 2	17.0 ± 2
	20	11.1 ± 2	14.8 ± 2	11.9 ± 2	16.4 ± 2

area 3 average pillar diameter		11.7 ± 1	15.2 ± 1	11.9 ± 1	16.6 ± 1

area 4	21	10.5 ± 2	12.6 ± 2	11.5 ± 2	17.5 ± 2
	22	10.3 ± 2	13.5 ± 2	11.6 ± 2	15.9 ± 2
	23	10.6 ± 2	12.9 ± 2	11.0 ± 2	15.6 ± 2
	24	10.2 ± 2	12.4 ± 2	10.5 ± 2	15.1 ± 2

area 4 average pillar diameter		10.4 ± 1	12.9 ± 1	11.2 ± 1	16.0 ± 1

area 5	25	9.6 ± 2	12.5 ± 2	10.6 ± 2	14.8 ± 2

In Figure 9a, for DCSCH, the proximity effect is clearly visible for pillars deposited with beam exposure times of 2 and 4 s. For a beam exposure time of 2 s, DCSCH pillars deposited in area 1 (Figure 7b) have an average diameter of 12.1 ± 1 nm, the average diameter of pillars deposited in area 2 is 12.4 ± 2 nm. Pillars deposited in area 3 have an average diameter of 11.7 ± 1 nm. In the second smallest area (area 4) the average diameter is 10.4 ± 1 nm, and the last deposited pillar (25 in Figure 7b) has the smallest diameter of 9.6 ± 2 nm. The average pillar base diameters for DCSCH pillars deposited in the various areas 1 to 5, with a beam exposure time of 4 s and a neighbouring pillar distance of 20 nm, are listed in Table 2. Similar square arrays were fabricated using SCH, as shown in Figure 9b. The upper set of pillars in Figure 9b was deposited with a beam exposure time of 2 s, and the distance between neighbouring pillars is 15 nm. The square array of pillars in the lower panel was deposited with a beam exposure time of 4 s, and the distance between neighbouring pillars is 20 nm. Measured base diameters of all pillars are tabulated in Table 2. The square array pillar deposition of SCH is analysed in the same manner as DCSCH. Based on the schematic shown in Figure 7b, the SCH square array pillar deposition shows five areas of different pillar diameters. The estimated average diameter of pillars deposited in area 1 is 12.5 ± 1 nm, in area 2 the average diameter is 13.1 ± 2 nm. Similarly in areas 3 and 4, the estimated average pillar diameters are 11.9 ± 1 and 11.2 ± 1 nm, respectively. The smallest base diameter pillar (area 5) obtained for SCH with a beam exposure time of 2 s and a neighbouring pillar distance of 15 nm is 10.6 ± 2 nm. The average diameters of the SCH square arrays of pillars deposited with a beam exposure time of 4 s and a neighbouring pillar distance of 20 nm, categorized into areas 1–5, are also included in Table 2. The broadening of the pillars in area 1 with respect to the pillar in area 5 results in a diameter increase of 2.5 ± 0.5 nm for DCSCH at a beam exposure time of 2 s and a neighbouring pillar distance of 15 nm. Similarly for a beam exposure time of 4 s and neighbouring pillar distance of 20 nm the diameter increase is 3.8 ± 0.5 nm. For SCH these numbers are 1.9 ± 0.5 nm and 2.2 ± 0.5 nm, respectively.

It is clear that both DCSCH and SCH show appreciable broadening through the proximity effect. However, considering the errors associated with the calculations of the relative broadening, the difference observed between DCSCH and SCH is not significant. Nonetheless, we find the relative broadening of deposits due to the proximity effect to be consistently more pronounced for DCSCH than for SCH. The difference between these compounds ranges from about 8% to about 40%, calculated as the increase in broadening when proceeding from SCH to DCSCH.

**Figure 10:**
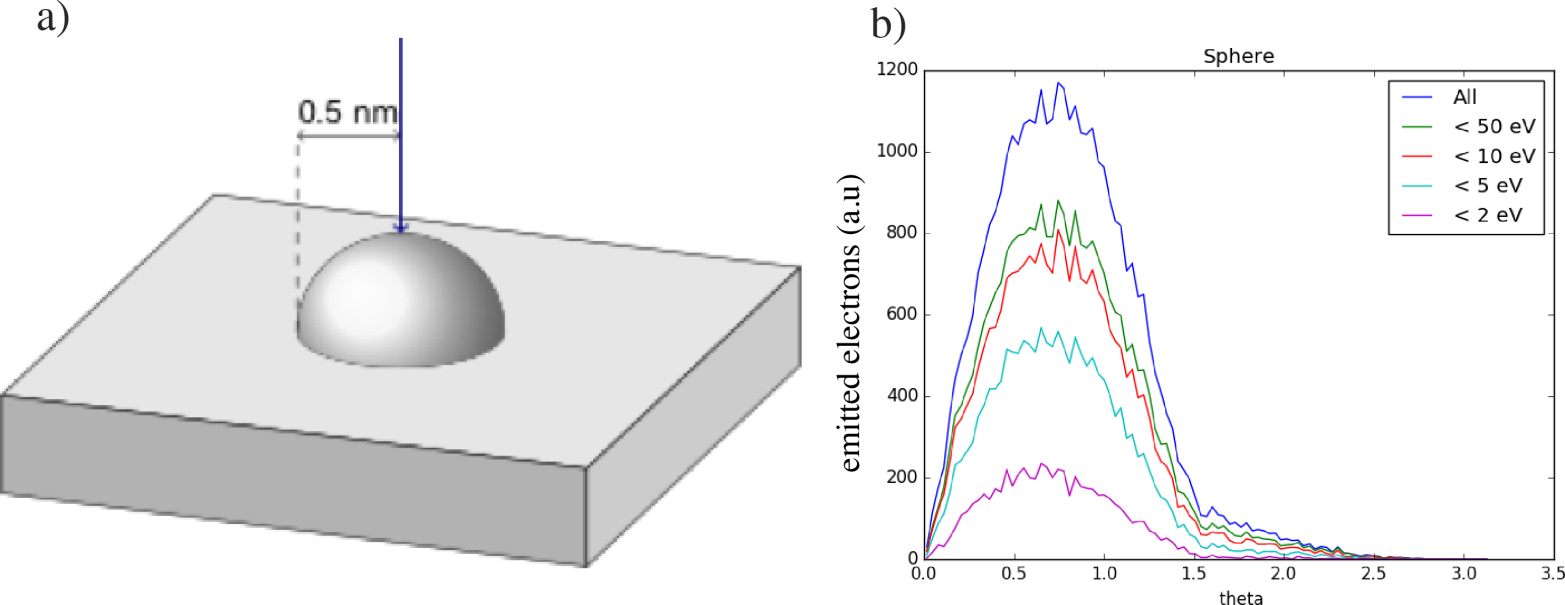
Monte Carlo simulation of the angular distribution of electrons emitted from a flat Si substrate with a 1 nm diameter Si half sphere on top (a), upon exposure with a “zero-diameter” 20 keV electron beam at the location of the top of the half sphere. b) The different curves are for emitted electrons with energy only below the indicated values. Theta is the angle between the emitted electron direction and the direction of the incident beam.

As discussed above, DEA to DCSCH is active for electrons of energies below 2eV and in the range from 6 to 9 eV. In order to contribute to deposit broadening through DEA, these low-energy electrons, generated in the growing pillar, need to reach the neighbouring dots. To obtain a better estimate of the number of low-energy electrons in the relevant energy range, a Monte Carlo simulation of the angular distribution of electrons escaping from a Si half sphere on the top of a Si surface was conducted. The sample for the simulation was a flat Si substrate with a 1 nm diameter Si half sphere on top, resembling a tiny Si deposit on a Si substrate (Figure 10a). The simulator contains the best possible physics models and runs on a GPU [48–49]. A “zero-diameter” 20 keV incident electron beam is directed on top of the half sphere, and subsequently all electrons emitted from the sample are recorded. For each emitted electron the energy, the direction and the location where it was emitted, is stored.

In Figure 10b the angular distribution of the electrons is shown, where the angle theta is the angle between the emitted electron and the incident electron beam. The top curve contains electrons of all energies up to 20 keV. The lower curves contain electrons of energy up to a maximum energy, as indicated in the figure. Integrating the intensity of electrons with energies below 2eV and in the range from 5 to 10 eV shows that the total number of electrons in this energy range is about 50% of the total number of electrons below 20 keV.

Considering the large number of secondary electrons emitted in the energy range relevant for DEA of DCSCH, and under the assumption that the DI cross sections are similar for both compounds, it is rather surprising that the difference between these compounds with respect to their relative broadening through the proximity effect is not more significant. However, as stated earlier, we do not know the absolute DEA or DI cross sections. Furthermore, these deposits are electrical isolators and during dot deposition, SEs and BSEs emitted from the growing dot may induce negative charges in neighbouring pillars. The trajectories of 2 eV electrons having to reach the neighbouring pillars may in turn be heavily influenced by the charging and may be prevented from reaching the pillar.

## Conclusion

Here we have presented the first study on electron beam induced deposition of SCH and DCSCH. We have characterized and compared the growth dynamics of these compounds and the composition of the deposits formed, as well as the extent of the proximity effect under different deposition conditions and geometrical deposition arrangements. We have further discussed the performance of these precursors in the context of their very different sensitivity towards DEA. That is, whereas DCSCH shows appreciable DEA cross sections, SCH is inert in this respect.

Fast initial lateral growth rates are observed for both precursors, but for DCSCH the lateral growth rate is found to be significantly higher than for SCH and the saturation diameter is about 30% larger for DCSCH as compared to SCH (90 nm as compared to 70 nm). Furthermore, in the early growth stage the volume growth rate of DCSCH is twice that of SCH. This meets the expectations that DEA should cause additional lateral growth and a higher volume growth rate. However, due to insufficient data on the absolute cross sections for these processes (DEA and DI) and for ND, as well as potential effects through the different chemical and physical properties of these molecules, we do not consider these results as conclusive in this respect. Potential approaches to achieve clearer differentiation between these two processes would be through targeted design of precursors with higher stability with respect to DI and increased sensitivity with respect to DEA. Such precursors should include predetermined breaking points where the DEA process is exothermic, as the attachment cross section is highest at threshold (close to 0 eV) and preferably the DEA process should lead to destabilization of the remaining moiety after the initial DEA process. In this context, the formation of hydrogen fluoride (bond dissociation energy (BDE) of about 6 eV [50]) has proven to increase molecular fragmentation through DEA considerably [51–52]. Combined with perfluorination of ligands to increase the attachment cross sections at threshold, this could be a viable approach to probe the relevance of DEA by very low energy (0+ eV) SEs produced by the primary electron beam in FEBID.

Composition analyses of the deposits by means of EDX reveal a close to stoichiometric Si/C ratio for both compounds and only a marginal amount of chlorine remains in the deposits formed with DCSCH. However, the Si/O ratio of the DCSCH deposits (2.4) is twice that observed for SCH deposits (1.2), indicating an efficient and complete formation of SiO_2_. We attribute this to an efficient hydrolysis of the Si–Cl bonds through residual water in the chamber and at the substrates surface. This is also consistent with the low chlorine content observed in the DCSCH deposits (desorption of HCl). Furthermore, such efficient hydrolysis of DCSCH is likely to reduce its susceptibility towards DEA by offering a competing channel for Si–Cl bond cleavage.

The proximity effect for both compounds is appreciable and although the relative broadening observed is consistently larger for DCSCH than for SCH, this difference is still within the accuracy of our measurements, and we do not consider this observation to give any conclusive information on the role of DEA, beyond that of DI in this process. According to the current Monte Carlo simulations, about 50% of all secondary electrons emitted below 20 keV falls within the energy range where DEA to DCSCH is active. Nonetheless, it is clear from this study that DI is very significant in the deposition of these precursors and the presence or absence of DEA is not a game changer in these cases. This may be due to efficient DI for both molecules blurring the additional effect expected due to the open DEA channels in DCSCH, or simply through a comparable total dissociation cross section of both compounds in the relevant secondary-electron energy range.

Independent of the ambiguity of the current results with respect to the role of DEA and DI, from this study there emerge two approaches worth exploring in the deposition of silicon containing nanostructures. First, the fact that the initial stoichiometric Si/C ratio is maintained in the deposit indicates that the deposition of silicon carbide may be achieved with a similar precursor with a higher Si/C ratio. We have identified the commercially available candidate trisilacyclohexane (TSCH) in which the stoichiometric Si/C ratio is 1:1, i.e., that of silicon carbide. We are currently studying this precursor. The oxygen content observed in the deposits of SCH, however, indicates that the formation of silicon carbide from the potential precursor TSCH might be further promoted through deposition under reductive conditions, e.g., in the presence of hydrogen. Secondly, the observable promotion of SiO_2_ formation from DCSCH, through hydrolysis of the Si–Cl bonds is a well-known process and might be purposely taken advantage of in the deposition of structurally intact SiO_2_ deposits.

## Experimental

The electron beam induced deposition and the inspection of the deposits was done in an FEI NovaNanoLab 650 dual beam scanning electron microscope. The precursor molecules were introduced into the SEM specimen chamber using a custom-built inlet system consisting of a stainless steel container with the precursor molecules and a leak valve. With the leak valve the pressure of the precursor molecules in the chamber could be precisely controlled.

The precursor dichlorosilacyclohexane (DCSCH, CAS No. 2406-34-0) was purchased from Gelest Inc, Morrisville PA, US. Silacyclohexane (SCH) was synthesized from DCSCH by following our reported procedure with a slight modification [35]. Briefly, a solution of 1,1-dichloro-1-silacyclohexane in diethyl ether was added dropwise to a lithium aluminium hydride solution (1.0 M in diethyl ether) at 0 °C and the mixture was stirred overnight at room temperature. The reaction mixture was then refluxed for three hours and excess LiAlH_4_ was quenched by treating with acidic solution (H_2_SO_4_). The organic layer was separated, dried over anhydrous Na_2_SO_4_ and filtered. The solvent was removed by distillation under reduced pressure to yield the crude product. The crude product was purified via condensation onto a liquid N_2_ cooled finger to yield the pure product and the analytical data matched with our previous report.

All deposition experiments were performed at room temperature. The background pressure of the system prior to deposition was (7–9) × 10^−7^ mbar. By leaking precursor molecules into the SEM chamber a precursor pressure was set to ca. 3 × 10^−5^ mbar for both precursor molecules. Both deposition and imaging were performed in ultra-high resolution mode, at a primary beam energy of 20 keV and a probe current of 150 pA. Pillar growth was achieved by spot exposure of the substrate at normal incidence. The primary electron beam exposure time and position was controlled using a stream file generated with the help of MATLAB. In all EBID experiments the working distance was 5.3 mm, close to the eucentric height of the system. The substrate material used for pillar deposition was silicon. All pillars were imaged top-down as well as under a tilt angle of 45°. Before imaging the deposits, the SEM chamber was pumped at least for 90 min after deposition to avoid unwanted further deposition due to remaining precursor molecules. This waiting time was sufficient to lower the background pressure to below 9 × 10^−7^ mbar. Pillar dimensions (height and base diameter) are estimated using the programme imageJ [53]. The base diameter was measured by fitting an ellipse to the base plane in the tilt images. The height was measured from the tilt images as well, and measured from the centre of the base plane to the apex of the deposit. The diameter of very shallow deposits arranged in arrays was measured by fitting circles to the perimeter of the deposits in the top-down SEM images. The pillar cone angle was measured by fitting straight lines to the edge of the cylindrical part of the pillar and the cone-shaped upper part using the imageJ programme [53]. The volumes of the pillars are estimated by approximating the pillar shape as a combination of a cylindrical lower part and a conical upper part of the pillar. From the estimated volume, the volume growth rate can be found by dividing the volume by the corresponding beam exposure time. The elemental composition of the deposited material was determined by energy-dispersive X-ray analysis of large volume deposits on gold substrates, using an Oxford Instruments 80 mm^2^ detector. EDX was performed at two different incident energies of 5 keV and 20 keV, at beam currents of 1.6 nA and 240 pA, respectively.
